# The effect of enteral nutrition strategy and non-invasive ventilation on diarrhea and nutritional goals in the critically ill: A protocol for a multicentre retrospective cohort study (ENND GOALS)

**DOI:** 10.1371/journal.pone.0322122

**Published:** 2025-04-22

**Authors:** Justin Bruni Senecal, Taiya Lakhanpal, Lawrence Mbuagbaw, Tatianna Giannidis, Parsa T. Khazaneh, Bram Rochwerg, Simon J.W. Oczkowski, Deborah J. Cook, Jennifer L.Y. Tsang, M. Ines Pinto-Sanchez, Sameer Sharif, David Armstrong, Natalia Ovtcharenko, Nicole Janisse, Tina Millen, Francois Lamontagne, Joanna C. Dionne

**Affiliations:** 1 Department of Medicine, Faculty of Health Sciences, McMaster University, Hamilton, Canada; 2 Department of Clinical Epidemiology and Biostatistics, McMaster University, Hamilton, Canada; 3 St. Joseph’s Healthcare Hamilton, McMaster University, Hamilton, Canada; 4 Department of Health Research Methods, Evidence and Impact, McMaster University, Hamilton, Canada; 5 Niagara Health Knowledge Institute, Niagara Health, Niagara, Canada; 6 Hamilton Health Sciences, Juravinksi Hospital, ICU, Hamilton, Canada; 7 Université de Sherbrooke, Department of Medicine, Sherbrooke, Canada; PLOS: Public Library of Science, UNITED KINGDOM OF GREAT BRITAIN AND NORTHERN IRELAND

## Abstract

Enteral nutrition (EN) has become the standard of care for nutritional support among critically ill patients. However, little is known about whether continuous delivery (CEN) or intermittent delivery (IEN) is preferable or the consequences of either strategy. This is particularly true for diarrhea, which is understudied but consistently shown to be associated with increased morbidity among critically ill patients. This is a multicenter, retrospective cohort study including critically ill patients greater than 18 years of age, admitted to the Intensive Care Unit in Hamilton, Canada, and prescribed EN for greater than 48 hours. Patients will be divided into IEN and CEN groups based on the nutritional strategy they received during their stay. The primary outcome will be the proportion of patients in each group with diarrhea during their ICU stay, diarrhea will be defined according to WHO criteria. Multivariate logistic regression will be performed to identify the role of covariates in the risk of developing diarrhea. Secondary outcomes will include caloric intake, incidence of ICU-acquired infections, including *Clostridioides difficile*, length of stay, and mortality. This study protocol has been approved by the Hamilton Integrated Research Ethics Board (#16453). The study findings will be disseminated at academic conferences and published in peer-reviewed journals.

## Introduction

Over the last thirty years, nutrition in the intensive care unit (ICU) has evolved from supportive therapy to an active treatment that can attenuate the harms of critical illness [1]. Early nutrition, delivered enterally, has become the standard of care for most critically ill patients and is recommended by the major organizations for clinical nutrition and critical care [1–4]. Enteral nutrition (EN) offers many physiological benefits throughout the gastrointestinal (GI) tract, including maintenance of intraepithelial junctions, mucosal lymphoid tissues, and villous height and lower infection risk by preventing bacterial translocation [5]. Furthermore, clinical studies have demonstrated a benefit with a recent meta-analysis by the European Society of Intensive Medicine (12 randomized controlled trials (RCTs), 597 patients) demonstrating a reduction in infectious morbidity when EN was delivered within 48 hours of ICU admission [3]. Another meta-analysis, including 6 RCTs and 5025 critically ill patients, showed a reduction in mortality with early EN compared to parenteral nutrition (PN) [4]. Despite this and guidelines recommending early initiation of EN, malnutrition in ICUs continues to be a problem with 56–68% of patients in Canadian ICUs being underfed relative to their calculated caloric requirements [6].

While guidelines agree on the value of early EN, there is little consensus on how feeds should be delivered [1,4]. Broadly, there are two methods for EN delivery: continuous enteral feeding (CEN), which consists of low rates of tube feeds over a 24-hour period, and bolus/intermittent enteral feeding (IEN), which consists of higher rates of tube feeds delivered over shorter time periods. The American Society of Parenteral and Enteral Nutrition (ASPEN) recommends avoidance of IEN in high-risk patients based on 2 small RCTs which showed a trend towards higher mortality with IEN (13.9% IEN vs 7.4% CEN; p = 0.18) and increased risk of ventilator association pneumonia with IEN (49.3% IEN vs 30.9% CEN, p=0.02) [1,7,8]. However, both trials were published more than 15 years ago. The European Society of Parenteral and Enteral Nutrition (ESPEN) recommends avoidance of IEN in general, citing trends towards worsening diarrhea in 5 small prospective studies [4]. In contrast, 2 meta-analyses, one including 5 RCTs (392 patients) and the other including 9 RCTs (690 patients) have shown no important difference in diarrhea, vomiting, nausea or mortality in patients fed via IEN vs CEN [9,10].

Just as the mode of EN delivery is understudied, so too are the effects of critical illness on the GI tract. Feeding intolerance, diarrhea, vomiting, and ileus are common and associated with increased mortality [11]. While challenging to define, a recent multi-center cohort study found the incidence of diarrhea was as high as 38% with various definitions [12]. Diarrhea has been consistently shown to increase length of stay and contribute to morbidity in ICU patients [11–12,14]. While EN in general has been associated with higher rates of diarrhea [13], the effect of EN strategy (IEN vs CEN) on diarrhea is still unclear. Two recent RCTs including 120 and 112 mechanically ventilated patients respectively found no significant difference in diarrhea, constipation or vomiting [15,16].

Taken together, EN has become the standard of care for most critically ill patients, but little is known about optimal EN strategy or its effect on diarrhea. This is a pragmatic retrospective cohort study that will compare patients receiving IEN and CEN by determining the differences in diarrhea incidence and nutritional exposures, such as the type and volume of EN that patients receive.

Our cohort will also capture patients receiving non-invasive positive pressure ventilation (NIPPV). NIPPV has become the standard of care for several indications and is integral for managing acute exacerbations of cardiopulmonary diseases [17]. There is a growing population of critically ill patients who remain on NIPPV for extended periods and whose optimum feeding strategy is unclear. As such, we will also describe the current nutritional strategies used for patients on NIPPV.

## Methods

### Design

We will conduct a retrospective multicentre cohort study, that evaluates the outcomes of patients with a prescription for enteral nutrition or are receiving NIPPV for >48 hours admitted to three academic ICUs in Hamilton, Ontario.

### Participants

We will include all patients 18 years of age or older admitted to the ICU for at least 24 hours who have a prescription for EN or are receiving NIPPV for greater than 48 hours. Patients who received majority NIPPV for 48 hours at any one time will also be included even if they were not prescribed EN. EPIC Hyperspace is now the Electronic Medical Record (EMR) used in hospitals in Hamilton. We will include patients admitted to the ICU since EPIC went live at each site. For St. Joseph’s Hospital Hamilton (SJHH) this is between January 1, 2018, and December 31, 2023. For the two sites at Hamilton Health Sciences (HHS), this is between June 4, 2022, and December 31, 2023. Exclusion criteria includes patients receiving only nocturnal NIPPV, patients who have had gastrointestinal surgery in the month preceding ICU admission, and patients who have confirmed or suspected luminal obstruction on admission. For the purposes of this study NIPPV will exclude high flow nasal canula (HFNC).

### Data collection

Data will be collected using the search function of EPIC Hyperspace and assistance from HHS and SJHH Information Technology (IT) services teams. The MRN numbers of patients meeting the inclusion criteria will be identified. The data will be exported directly to excel. Any missing data from IT reports will be entered via manual chart review. For each recruited patient, the following baseline characteristics will be recorded to determine factors associated with intolerance of IEN or CEN and with the development of ICU-acquired diarrhea. The baseline characteristics will include demographic data, anthropometric data, clinical data on Day 1 of ICU stay (surgical, medical vs trauma admission, diagnostic category, admitting diagnosis, ventilation status, inotrope/ vasopressor use, renal replacement therapy), admitting hospital, medical comorbidities (prior GI disease, previous luminal surgeries), nutritional assessment (caloric goals on admission will be determined using weight and registered dietician assessment), antibiotic prescription on admission and serum albumin on ICU admission. Table 1 outlines the primary and secondary outcomes as well as the data that will be recorded each day of the ICU admission up until the 30th day or ICU discharge. This includes mode of nutrition (IEN, CEN, oral, or PN), daily caloric intake, mode of ventilation (Invasive, NIPPV), presence of diarrhea, bacteremia greater than 48 hours after admission (gram-positive bacilli and coagulase-negative staph will be reviewed and omitted if team feels it is more likely contaminant), incidence of aspiration pneumonia/pneumonitis, *Clostridioides difficile* infection (CDI), presence of vomiting, ileus or abdominal distention, vasopressor/inotrope dose, average daily insulin dose and blood glucose, survival status in ICU, ICU length of stay. Survival to hospital discharge and hospital length of stay will be collected for the entire hospital stay.

**Table 1:** Statistical Plan.

Research Objectives	Outcome	Covariates/Subgroups	Analytical Approach
To determine the incidence of diarrhea amongst patients on IEN and CEN	WHO-defined diarrhea	N/A	Descriptive statistics (proportion of patients with diarrhea) and corresponding 95% CI
To determine risk factors associated with diarrhea during ICU admission	**Dependent Variable** WHO-defined diarrhea	Age, sex, type of nutrition (IEN/CEN), antibiotics, GI comorbidities, vasopressor/inotropes, daily caloric intake, NIPPV, surgical/trauma/medical admission	Logistic Regression
Secondary Outcomes			
**Research Objectives**	**Outcome**	**Covariates/Subgroups**	**Analytical Approach**
To determine whether IEN or CEN is better for reaching caloric requirements during ICU stay	**Dependent Variables:** Average caloric intake (percent goal determined by RD or 25 kcal/kg) at 48 hours and 7 days after initiation of CEN/IEN Proportion of patients on PN at 48 hours and 7 days after initiation of CEN/IEN	NIPPV, Age, sex, antibiotics, GI comorbidities, type of nutrition (IEN/CEN) vasopressor/inotropes, daily caloric intake, surgical/trauma/medical admission	Descriptive statistics (average caloric intake) and corresponding 95% CI Descriptive statistics (proportion of patients on PN) and corresponding 95% CI Logistic regression
To determine whether IEN or CEN is associated with ICU-acquired bloodstream infection or *Clostridium difficile* infection	Incidence of positive blood cultures drawn at least 48 hours after admission, excluding coagulase-negative staphylococcus unless suspected by ICU team to not be contaminant.	NA	The incidence of bloodstream infections will be computed as the number of new cases during ICU stay divided by the person-time at risk. Descriptive statistics (proportion of patients with ICU-acquired bloodstream infection or *C. difficile infection*) and corresponding 95% CI
To determine whether IEN or CEN is associated with differences in mortality or length of stay (ICU or hospital)	**Dependent Variables** Mortality (ICU and hospital) Length of stay (ICU and hospital)	Age, sex, type of nutrition (IEN/CEN), antibiotics, GI comorbidities, vasopressor/inotropes, daily caloric intake, NIPPV, surgical/trauma/medical admission	Descriptive statistics (mortality and length of stay) and corresponding 95% CI Logistic regression
To determine whether IEN or CEN is associated with differences in aspiration pneumonia/pneumonitis	Incidence of aspiration pneumonia/pneumonitis, determined pragmatically by ICU team	NA	The incidence of aspiration pneumonia/ pneumonitis will be computed as the number of new cases during ICU stay divided by the person-time at risk. Descriptive statistics (proportion of patients with aspiration pneumonitis/ pneumonia) and corresponding 95% CI

### Outcomes

The primary outcome is the incidence of diarrhea defined according to the WHO definition (greater than 3 liquid bowel movements in 24 hours) any time during the index ICU admission (Table 1). This will be identified from nursing flowsheets in EPIC. The secondary outcomes include mortality (ICU and hospital), length of stay (ICU and hospital), incidence of confirmed or suspected hospital-acquired blood stream infections or central line associated blood stream infections, incidence of aspiration pneumonia/pneumonitis (identified pragmatically as documented by the ICU team), CDI, percentage of caloric requirements received by enteral nutrition (daily caloric requirements defined as 25 kcal/kg/day1 and by registered dietician assessment), incidence of vomiting, ileus and abdominal distention, and glycemic control (using average daily insulin dose and average daily glucose as a surrogate) (Table 1).

### Sample size

As this is a retrospective study, our sample is dependent on the number of patients who are eligible and classified in each of the IEN and CEN groups. On initial review of the EMR at each site, we expect that approximately 500 patients at HHS and SJHH meet the inclusion criteria. We suspect that the number of patients receiving IEN will be less than that of those receiving CEN.

A recent meta-analysis of observational studies including 717 patients across 11 studies found that 25.1% of patients on IEN developed diarrhea while only 17.5% on CEN had diarrhea [18] Using a two-tailed z-test approximation of independent proportions with a power 0.8 and alpha of 0.05, we estimate that each group would require 455 patients [19]. Given this calculation we suspect that the study will be adequately powered for the primary outcome of interest.

### Pilot testing

We will begin with an iterative process of 40 randomly selected patients, 20 from each site. This will allow us to review and refine the reports generated by the IT service teams. Each of the 40 charts will be adjudicated by two reviewers to determine the accuracy of IT reports. We will adjudicate on the primary and secondary outcomes, and key clinical data including mode of nutrition, timing of nutrition, and mode of ventilation. This will operationalize the suitable patient identification for this study, identify any challenges needing to be addressed before embarking on the study in the operations or case report forms and it will help to enhance the validity of our data. Once optimized we will only manually review charts for missing/unclear data.

### Data adjudication

To assure the accuracy of our data, a second round of adjudication after the pilot exercise and after data collection will be performed. A total of 50 patients will be randomly selected from SJHH and HHS (25 from each site). Each of the charts will be adjudicated by two adjudicators. Adjudication will again be performed as explained above for pilot testing. This data will be compared to that generated from reports to ensure agreement.

### Data analysis

The data analysis will include both inferential and descriptive statistics. For patient characteristics the patient data from the medical record will be summarized descriptively with categorical variables expressed as percentages. The percentages and quantitative variables will be expressed as means with standard deviation or medians with inter quartile range when appropriate. Comparisons between groups will be made via Chi-square test.

Table 1 includes the primary and secondary outcomes statistical plan. For the primary outcomes patients will be divided into two groups based on exposure to IEN. Patients receiving IEN (as specified in EPIC orderset) for 2 complete days and most of the study days will be placed in the IEN group will remain in the CEN group. The proportion of patients in each group with ICU acquired diarrhea with associated 95% Confidence Interval (CI), will be reported as a primary outcome. Univariate and multivariate logistic regression will be used to evaluate the role of covariates on the risk of developing diarrhea during the ICU admission (Table 1). Results will be reported as odds ratio (OR) with 95% CI.

For the secondary outcomes patients also be divided into those who have received NIPPV and those who have not. For the purposes of this study, NIPPV will exclude HFNC but will include bilevel positive airway pressure (BiPAP) and continuous positive airway pressure (CPAP) delivered via any interface. To evaluate the relationship between IEN, CEN and NIPPV on caloric goals, each patient’s caloric intake at 48 hours and 7 days will be divided by their 24 hour caloric target (as determined by RD assessment and by the algorithm 25 kcal/kg) (Table 1). The average of these percentages with associated 95% CI will be calculated in each group. The proportion of patients in each group receiving supplement parenteral nutrition at these time points will also be determined to help evaluate the effectiveness of each EN strategy (Table 1). Logistic regression will also be applied to evaluate the role of covariates; specifically baseline characteristics and the effect they may have on patients reaching their caloric targets (Table 1). In terms of other secondary outcomes, the incidence of hospital acquired infections, ICU and hospital mortality will be determined per person-time at risk in each group (IEN vs CEN) with associated 95% CI. Significant differences between groups will again be determined by two sample t-test. Multivariate logistic regression will evaluate associations between baseline characteristics and the type of EN chosen.

### Feasibility

Given the broad inclusion criteria, we expect to have many eligible patients for this study. The feasibility of this study relies on the ability to generate reports from the EMR to avoid manual chart review when possible. We have involved IT teams from each of the sites and have confirmed that most of the data we seek can be exported directly from EPIC hyperspace to Microsoft Excel. Specifically, they have confirmed that data recorded in the EPIC flowsheets, including ventilatory support (including NIPPV), bowel movements, and vasopressor dose can be transported directly to excel. Other data, including EN prescription (including IEN vs CEN), PN prescription, microbiology, glucose, and insulin dosing, are readily accessible. This data can be sorted directly into Excel documents without the need for manual chart review. This leaves only some admission data and incidence of aspiration pneumonia that will require manual chart review. This will not be required for all our primary and most of our secondary outcomes.

### Timeline

Access to patient charts was gained on Dec. 21, 2024. Screening records is underway, and we expect this to be completed by April 30, 2025. Data extraction should be completed by July 2025. We expect data analysis to be completed by Dec. 2025.

### Ethical and dissemination

This study has been approved by the Hamilton Integrated Research Ethics Board (#16453). Our request for waiving informed consent, given the retrospective nature of our study, has been approved. Access to patient records has been approved by both HHS and SJHH. Identifying information (patient ID number, age, and date of admission) has been provided by the hospitals, as manual review may be required for data clarification. Once collected patient data will be anonymized. No patient will be exposed to any inconvenience as all data will be retrospective. Only members of the study team will have access to patient records with an identification number being assigned to each patient to anonymize data.

The study findings will be disseminated at academic conferences and published in peer-reviewed journals.

## Discussion

EN has become the standard of care for nutritional support in most critically ill patients. Despite this, nutrition and the consequences of delivering it are discussed by multidisciplinary teams and often family members daily in the ICU. Abdominal pain, distention, vomiting, and diarrhea are frequent [11], and there is little to no high-quality evidence outlining which EN strategies best mitigate these risks. Institutional practices and practitioners’ comfort with specific strategies often dictate the EN method.

Diarrhea is a frequent problem amongst critically ill patients that is also understudied. It has been shown in a recent multicentre prospective study to affect greater than 38% of critically ill patients [12]. Moreover, diarrhea is particularly distressing to patients and their families and has been shown in several studies to be associated with increased length of stay. Selecting an EN strategy that would be less likely to result in diarrhea would be valuable to clinicians and patients alike.

Physiological studies have suggested the potential benefits of IEN over CEN [20]. This is despite CEN being more common than IEN in most critical care units [20]. In healthy volunteers, IEN causes more rapid gastric emptying, increased superior mesenteric arterial flow, and physiologic insulin peaks compared to CEN [20]. While caution should be used when generalizing these findings to critically ill patients, a small RCT of 120 patients did find that mechanically ventilated patients on IEN were more likely to meet caloric targets. Furthermore, interrupted feeds for procedures and loss of enteral access are more likely to affect the daily caloric intake amongst patients on CEN. With malnutrition still being a large problem in Canadian ICU, IEN may be a potential solution and led us to evaluate the effects of EN strategy on the daily caloric intake and PN [21]

One group of ICU patients in whom the question of IEN vs. CEN is crucial is patients receiving NIPPV. NIPPV can reduce the need for invasive ventilation in patients with hypercapnic respiratory failure and cardiogenic pulmonary edema [17] However, delivering optimum nutritional support is challenging in patients receiving NIPPV. Fears regarding EN initiation in those receiving NIPPV may be related to the reported risk of airway complications [22]. In a retrospective cohort study of 107 patients, those receiving EN while on NIPPV had higher rates of mucus plugging (50% vs 30%; p=0.035) and aspiration pneumonia (17% vs 4%; p=0.04) compared to those not receiving EN [22]. Delivery of EN may also limit the effectiveness of NIPPV by causing gastric dilatation or air leaks associated with concurrent use of a feeding tube and NIPPV device [23]. Understanding trends in the nutritional care of patients on NIPPV is crucial and led us to include patients on prolonged courses of NIPPV irrespective of their receipt of EN.

### Strengths and limitations

This is a pragmatic study with broad inclusion criteria that should be generalizable to most critically ill patients. It uses the newly implemented EMR at all institutions in Hamilton to generate a large amount of patient data without manual review. It examines two understudied areas of critical care medicine, the effects of EN strategy on diarrhea and the effect of NIPPV on nutrition.

The main weakness in our study is the retrospective strategy. It may be challenging to understand whether differences in diarrhea and daily caloric intake are due to covariates and the severity of illness or the EN strategy chosen. Furthermore, our study centres are all located in Hamilton, Canada; meaning that our findings may be less generalizable to other centres with different practice patterns.

The ENND-GOALs study will generate relevant, pragmatic data regarding EN strategy and diarrhea in the critically ill. Moreover, it will support the findings and inform posthoc analysis from the upcoming INVENTS-ICU RCT, which will also evaluate the differences between IEN and CEN.
